# A Peer-to-Peer Live-Streaming Intervention for Children During COVID-19 Homeschooling to Promote Physical Activity and Reduce Anxiety and Eye Strain: Cluster Randomized Controlled Trial

**DOI:** 10.2196/24316

**Published:** 2021-04-30

**Authors:** Yingfeng Zheng, Wei Wang, Yuxin Zhong, Fengchun Wu, Zhuoting Zhu, Yih-Chung Tham, Ecosse Lamoureux, Liang Xiao, Erta Zhu, Haoning Liu, Ling Jin, Linyi Liang, Lixia Luo, Mingguang He, Ian Morgan, Nathan Congdon, Yizhi Liu

**Affiliations:** 1 State Key Laboratory of Ophthalmology Zhongshan Ophthalmic Center Sun Yat-sen University Guangzhou China; 2 Guangzhou Regenerative Medicine and Health Guangdong Laboratory Guangzhou China; 3 Research Units of Ocular Development and Regeneration Chinese Academy of Medical Sciences Guangzhou China; 4 Department of Psychiatry Affiliated Brain Hospital of Guangzhou Medical University (Guangzhou Huiai Hospital) Guangzhou China; 5 Department of Psychiatry Guangdong Engineering Technology Research Center for Translational Medicine of Mental Disorders Guangzhou China; 6 Singapore Eye Research Institute Singapore National Eye Centre Singapore Singapore; 7 Zhaoqing Education Bureau Zhaoqing China; 8 Duanzhou District Education Bureau Zhaoqing China; 9 Research School of Biology Australian National University Canberra Australia; 10 Centre for Public Health Queen’s University Belfast Belfast Ireland

**Keywords:** homeschooling, children, anxiety, digital eye strain, peer to peer, live streaming, digital health, intervention, health information, physical activity, COVID-19, online learning, behavior, app, mobile phone

## Abstract

**Background:**

The COVID-19 pandemic has led to worldwide school closures, with millions of children confined to online learning at home. As a result, children may be susceptible to anxiety and digital eye strain, highlighting a need for population interventions.

**Objective:**

The objective of our study was to investigate whether a digital behavior change intervention aimed at promoting physical activity could reduce children’s anxiety and digital eye strain while undergoing prolonged homeschooling during the COVID-19 pandemic.

**Methods:**

In this cluster randomized controlled trial, homeschooled grade 7 students at 12 middle schools in southern China were recruited through local schools and randomly assigned by the school to receive (1:1 allocation): (1) health education information promoting exercise and ocular relaxation, and access to a digital behavior change intervention, with live streaming and peer sharing of promoted activities (intervention), or (2) health education information only (control). The primary outcome was change in self-reported anxiety score. Secondary outcomes included change in self-reported eye strain and sleep quality.

**Results:**

On March 16, 2020, 1009 children were evaluated, and 954 (94.5%) eligible children of consenting families were included in the intention-to-treat analysis. Children in the intervention (n=485, 6 schools) and control (n=469, 6 schools) groups were aged 13.5 (SD 0.5) years, and 52.3% (n=499) were male. The assigned interventions were completed by 896 children (intervention: n=467, 96.3%; control: n=429, 91.5%). The 2-week change in square-root–transformed self-reported anxiety scores was greater in the intervention (–0.23, 95% CI –0.27 to –0.20) vs control group (0.12, 95% CI 0.09-0.16; unadjusted difference –0.36, 95% CI –0.63 to –0.08; *P*=.02). There was a significant reduction in square-root–transformed eye strain in the intervention group (–0.08, 95% CI –0.10 to 0.06) compared to controls (0.07, 95% CI 0.05-0.09; difference –0.15, 95% CI –0.26 to –0.03; *P*=.02). Change in sleep quality was similar between the two groups.

**Conclusions:**

This digital behavior change intervention reduced children’s anxiety and eye strain during COVID-19–associated online schooling.

**Trial Registration:**

ClinicalTrials.gov NCT04309097; http://clinicaltrials.gov/ct2/show/NCT04309097

## Introduction

COVID-19 has spread worldwide, with more than 24 million confirmed cases as of August 29, 2020 [1]. UNESCO (United Nations Educational, Scientific and Cultural Organization) announced that at least 188 countries have closed schools nationwide as of August 2020, which has resulted in the unprecedented adoption of online homeschooling [2]. An estimated 1.5 billion children are learning at home without direct access to school activities [2].

Prolonged adoption of homeschooling has important implications for children’s mental health. Although the long-term mental health consequences of the COVID-19 pandemic on children have not been systematically explored [3-5], it has been suggested that quarantined children have an average posttraumatic stress score 4 times higher than nonquarantined children [6]. Given that school isolation and stay-at-home orders could further worsen anxiety and other mental health problems, there is a critical need for novel interventions to safeguard the well-being of students in this regard [7-9].

In addition to its psychological impacts, the COVID-19 pandemic can take a toll on children’s vision as many schools move online. The expanded use of digital devices and increased screen time may result in worsening symptoms of eye strain and pose greater risk for myopic progression [10]. The American Academy of Ophthalmology has recommended the 20-20-20 rule, which calls for taking a visual break every 20 minutes by looking at an object 20 feet away for 20 seconds. While it is important to deliver such health information, it is equally necessary to study effective ways to promote behavior change, motivate eye relaxation exercises, and test effectiveness in reducing eye strain.

Digital behavior change interventions have been considered as an effective approach to promote physical activity and/or reduce sedentary behavior [11]. Furthermore, physical activity may be a useful target for strategies to handle stress, anxiety, sleeping problem, and eye relaxation, given previous reports regarding the associations of a lower level of physical activity with anxiety [12], depression [13], sleep disorder [14], and dry eye disease [15]. We have therefore hypothesized that a digital behavior intervention aimed at promoting physical activity could improve outcomes in anxiety, sleeping, and eye strain. This issue is critically important, given the magnitude of the COVID-19 pandemic. While this manuscript was under review, it was reported that individuals with inadequate physical activity reported a greater level of psychological distress during the COVID-19 pandemic [16].

The advent of digital technology has provided new opportunities to deliver digital behavior interventions on a population scale [17]. More than 90% of American teenagers are online every day (averaging more than 6.5 hours daily), where they spend much of their time interacting with friends and family through social media [18]. More than 40% of adolescents in grades 7 to 9 in China report at least 2 hours of exposure to digital screens per day [19]. In particular, live-streaming apps have become a popular form of social entertainment for children. However, despite the increasing adoption of live-streaming apps among children and teenagers, their use in public health interventions has yet to receive wide attention in clinical trials.

In light of this gap, we developed a novel digital behavior change intervention that encourages children to engage in regular physical activity and relaxation of accommodation (near focusing) during online school recess periods. The aim of this study is to evaluate the effectiveness of this digital intervention in reducing anxiety syndrome (main outcome) and eye strain, compared to a conventional educational intervention among Chinese children during a recent period of home confinement occasioned by the COVID-19 pandemic.

## Methods

### Study Design and Participants

This cluster randomized controlled trial was conducted in the Duanzhou district of Zhaoqing City, Guangdong Province, in southern China. This setting was chosen because Zhaoqing, one of southern China’s major cities, is readily accessible from Guangzhou, the provincial capital. The 2016 national census shows that Zhaoqing has a relatively stable population of 4,084,600, which is representative of the Chinese national urban population in terms of demographic and socioeconomic characteristics [20]. Smartphone use is very widespread in this area, as throughout the rest of China, where the number of mobile phone subscribers in December 2019 (1.6 billion) exceeded the total population of 1.43 billion [21]. China has mandated 9 years of compulsory education since 1986, and the enrollment rate for the 7th grade (12-14 years) is nearly 100% in Zhaoqing. Thus, the school-going cohort is representative of children in the general population.

The research protocol was approved by the Institutional Review Board of the Zhongshan Ophthalmic Center, Sun Yat-sen University, and the study was performed in accordance with the Declaration of Helsinki. Written informed consent was obtained from at least one parent or guardian of all participating children. The date of registration of the clinical trial was March 16, 2020.

The COVID-19 outbreak was recognized in China during the nationwide winter school vacation. On January 27, 2020, China’s Ministry of Education announced the postponement of the 2020 spring semester, and all schools were asked to recommend that students stay at home, avoiding group activities and large gatherings. As the outbreak continued, the Guangdong Education Department announced in February 2020 that secondary schools would begin formal online education beginning March 2, 2020. Teachers at the 14 public secondary schools in Duanzhou district (Figure S1 and Table S1, Multimedia Appendix 1) formulated an online curriculum for homeschooling, with standardized content and schedules for all students in the area (Table S2, Multimedia Appendix 1).

After excluding 2 secondary schools owing to an insufficient number of students per cluster, permission to conduct the study was requested from the remaining public secondary schools (n=12) in the district (Figure S1, Multimedia Appendix 1). Inclusion criteria were as follows: (1) grade 7 (12-13 years old) students in Duanzhou district and (2) under home confinement and enrolled in online learning courses during the COVID-19 outbreak. Exclusion criteria included the presence of disorders such as autism, pervasive developmental delay, and schizophrenia, which might interfere with participation in the intervention. The study followed the CONSORT (Consolidated Standards of Reporting Trials) guideline [22] (Multimedia Appendix 2). The reporting of the mobile-phone–based questionnaires followed the CHERRIES checklist (Checklist for Reporting Results of Internet E-Surveys) [23] (Multimedia Appendix 3).

### Randomization, Concealment, and Masking

Cluster randomization with schools as the cluster was adopted in order to maximize peer-to-peer support while avoiding contamination in the use of social media between study groups. A total of 12 schools with a block size of 4 were assigned (1:1) to receive either health education + download of the peer-to-peer live-streaming app (intervention group) or health education only (control). The randomization sequence was generated by an independent statistician using an online random number generator. In each school, 2 classes (on average, 40 students per class) were further randomly selected for participation.

Due to the nature of the intervention, students were not masked. However, masking of investigators was achieved through the exclusive use of electronic, self-administered questionnaires and masking the statistician to group allocation until completion of all analyses. In addition, participating students were informed that their responses to questionnaires would not be made available to their parents or teachers. Qualitative feedback on the app was not obtained from participants.

### Procedures

Recruitment and enrollment were assisted by trained teachers, who described the study in detail to participants and their parents over the phone. Participants and their parents were offered the opportunity to ask questions about participation. Before randomization, online seminars for parents were conducted through all 12 participating schools, during which the investigators answered questions and collected consent forms. Teachers were trained and were provided with a manual of operations, which included the study protocol and standard operating procedures for the study.

During the period of home confinement, the Chinese government had already issued a policy recommending a specific schedule for recess and physical activity breaks for students. In the control group, teachers delivered an online health information session covering the following topics and health advice to students: (1) an outline was provided on the recommended 20-20-20 rule during study and viewing of on-screen content; (2) during recess (15 mins for each recess; 4 times per day), participants in the control group received SMS text message prompts (≤50 characters) to participate in broadcast exercise programs at home, eye relaxation, or to stretch for 10 minutes. Students had access to at-home workout videos developed by exercise physiologists. Breaks were part of the online curriculum, and students were instructed by teachers (who were not aware of the study allocation) to rest and take exercise breaks according to government-issued recommendations.

Students in the intervention group received the identical health information session, online curriculum, workout videos, and breaks as described above. Additionally, at the beginning of the study, students in the intervention group were asked to log on and download a peer-to-peer live-streaming app (the Recess and Exercise Advocacy Program [REAP]). REAP is a live-streaming platform that allows users to capture short videos and photographs with their smartphones related to their physical exercise or eye relaxation activities (eg, looking outdoors through the window). The app has been optimized for both iOS (Apple) and Android operating systems. During each recess from homeschooling (15 mins for each recess; 4 times per day), participants in the intervention were prompted by SMS text messaging (≤50 characters) to log in to the REAP app to participate in live streaming or posting their workouts and outdoor videos and photographs (Supplementary Method S1, Multimedia Appendix 1).

The outcomes were measured by a self-assessed survey through mobile-phone–based questionnaires after signing the informed consent form. The questionnaires were surveyed at the beginning of the study and at the 2-week follow-up. At the start of the questionnaire, participants were given the following information: the purpose of the study, the expected length of time of the mobile phone survey, the names of the investigators, and the way and duration the data were stored. To ensure completeness checks, the participants were asked to complete the mandatory items before the questionnaire was submitted. The study investigators ensured that the confidentiality of the participants’ data was preserved. Individual participant data will not be disclosed to outside personnel and will not appear in any publications. Data assistants entered the questionnaires into a database and pseudonymized the data before it passed to the study statistician.

### Outcome Measures

The primary study outcome was a change in self-reported anxiety score between baseline and the 2-week follow-up, as measured using the 45-item Chinese version of the Spence Children’s Anxiety Scale (SCAS). In addition to the total SCAS score, this measure assesses 6 domains of anxiety: panic/agoraphobia, separation anxiety, social phobia, fears of physical injury, obsessive compulsive problems, and generalized anxiety. Details relating to the SCAS can be found in Multimedia Appendix 1 [24]. This instrument has been well validated in Chinese children [25]. A higher total score (range 0-114) summing the point values of all items indicates greater anxiety.

Secondary outcomes included changes from baseline to the 2-week follow-up in the following items: children’s self-reported eye strain, sleep quality, and time spent on different near-work activities. Eye strain was measured with the self-reported Computer Vision Syndrome Questionnaire (CVS‐Q), which evaluates the frequency (never, occasionally, or often/always) and intensity (moderate or intense) of 16 symptoms. Details relating to the CVS-Q scale are available in Multimedia Appendix 1 [26]. The 4-item Patient-Reported Outcomes Measurement Information System (PROMIS) pediatric sleep disturbance questionnaire was used to assess sleep disturbance (Multimedia Appendix 1) [27]. Self-reported information regarding the average daily time spent on near-work activities was collected, including each of the following: reading; writing; and use of computers/tablets, smartphones, television, and video games (Multimedia Appendix 1).

In addition, parentally reported anxiety scores on behalf of children were obtained using the SCAS for Parent (SCAS-P) questionnaire (Multimedia Appendix 1). This variable was not prespecified but was used to assess the reliability of self-reported scores from children.

### Statistical Analysis

The sample size was calculated based on a cluster randomized design, assuming that children’s self-reported raw anxiety score in the intervention group would be reduced by 2.5 points, with a standard deviation of 5 points, and the anxiety score of the control group would not change. The average cluster size was about 80, and an intracluster correlation coefficient of 0.02 was estimated based on the Refractive Error Studies in Children [28]. A sample size of 8 schools (4 in each group) at a two-sided significance level of .05 would give a power of 90%. Assuming a participation rate of 90% and attrition of 20%, a total of 12 schools was required. The sample size was calculated using PASS 16.0 (NCSS, LLC).

The distribution of baseline characteristics was presented as the mean (SD) for continuous variables and frequency (percentage) for categorical variables. The unadjusted mean differences between study groups in 2-week change and the 95% CI for primary and secondary outcomes were calculated using linear regression. The adjusted intervention effect on the primary outcome and 95% CI were estimated using linear regression models, adjusting for baseline measures. The study group and all variables with *P*<.20 in univariable regression analyses were included in the multivariable regression analysis. Histograms and quantile-quantile plots were used to verify the normality assumptions of the *t* test and linear regression models. Square-root transformation was applied to all outcomes due to lack of normality. To satisfy intention-to-treat criteria, we conducted missing data imputation. Multiple imputation was used to create 20 copies of the data, and final results were obtained by averaging these data sets using the rules developed by Rubin [29]. A two-sided *P* value less than .05 was considered statistically significant. All analyses were performed using Stata 15.0 (Stata Corp). The study protocol was registered at ClinicalTrials.gov (NCT04309097) prior to enrollment of the first participant and is available for online access.

### Data-Sharing Statement

Requests for anonymized individual participant data and study documents will be considered on a case-by-case basis and on scientific merit by the principal investigators and approved by the relevant institutional review boards.

## Results

**Figure 1:**
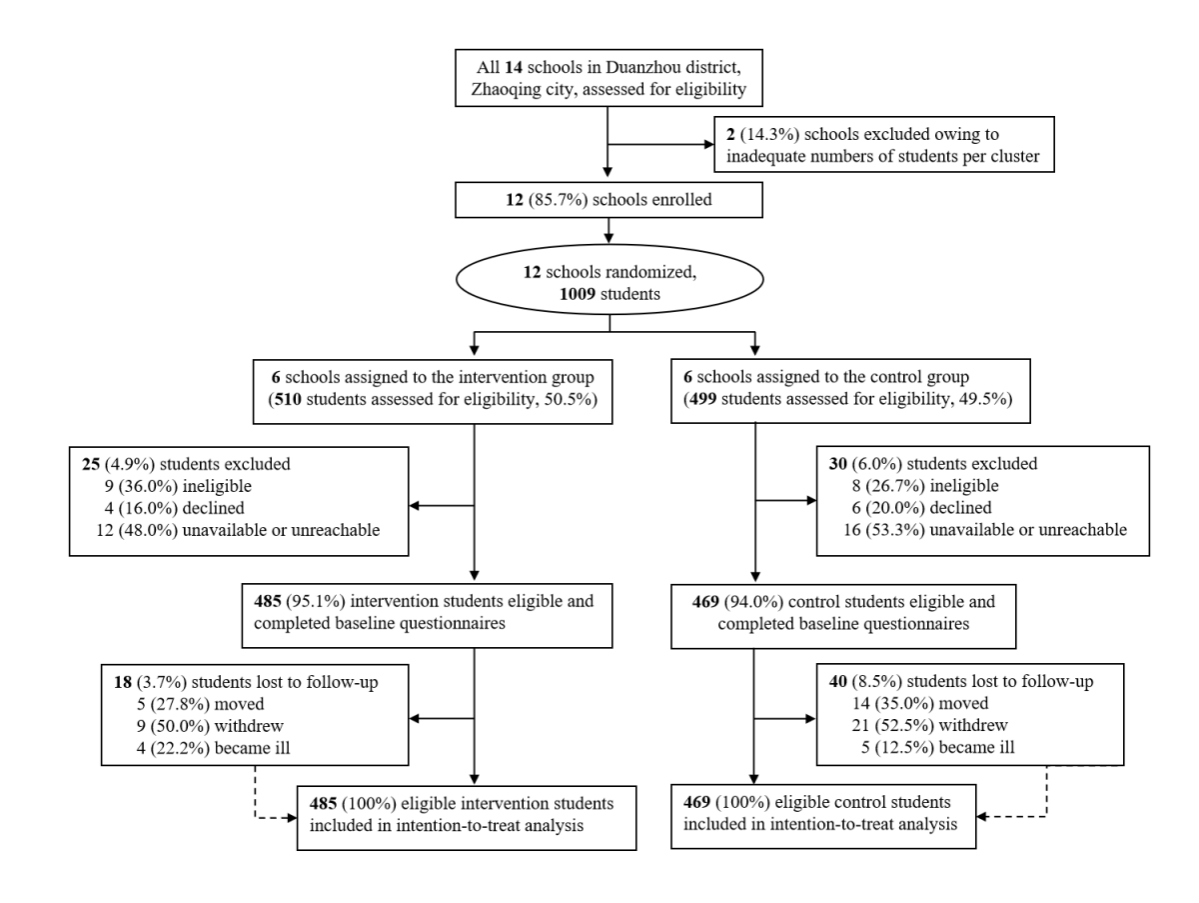
Flow diagram for the trial.

A total of 12 schools were randomized (6 to the intervention group and 6 to the control group; Figure 1). Of the 1009 grade 7 students assessed for eligibility (intervention group: n=510, 50.5%; control group: n=499, 49.5%), 55 (5.45%) were unreachable, ineligible, or declined to participate, leaving 954 (94.5%) eligible students, with 485 (95.1%) in the intervention group and 469 (94.0%) as controls. Rolling recruitment occurred on March 16, 2020. During the 2-week follow-up period, 18 (3.7%) and 40 (8.5%) students withdrew from the intervention and control groups, respectively (Figure 1).

The baseline characteristics of students in the study groups were similar (Supplementary Table S4, Multimedia Appendix 1). The mean age was 13.5 (SD 0.50) years in both groups, and there were no significant baseline differences in sex, use of glasses, parental education and smoking, and family income.

**Figure 2:**
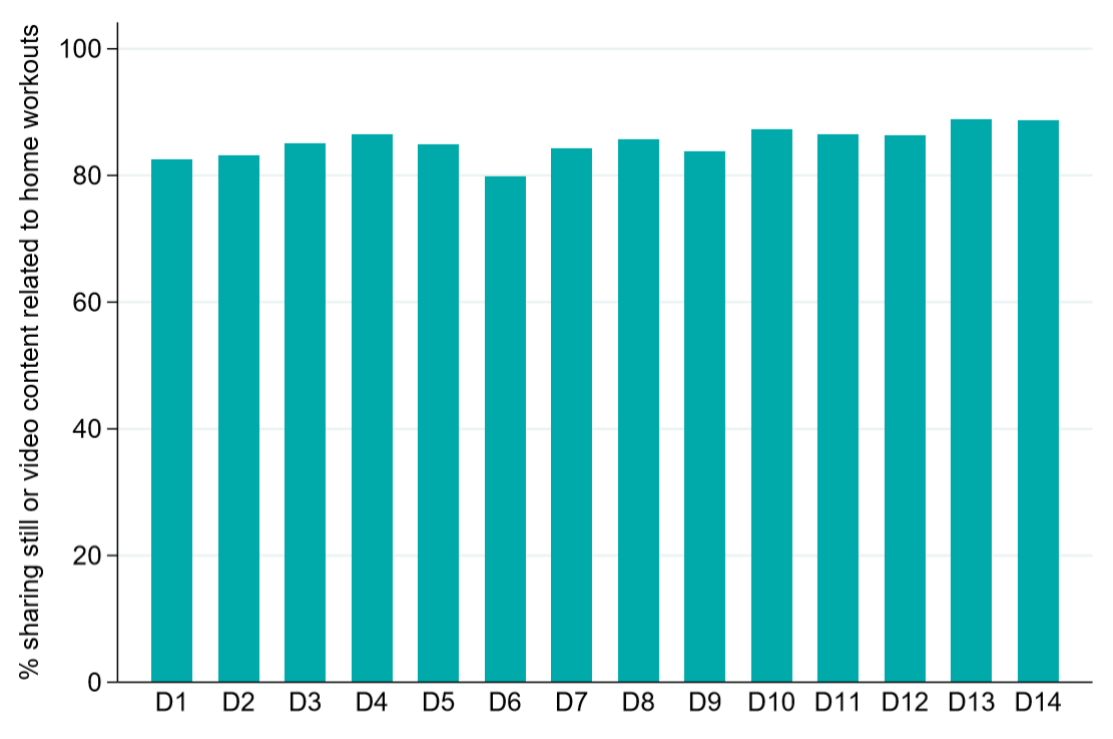
The proportion of participants sharing photos or video content related to home workouts in the intervention group.

Intervention compliance was monitored using the Cloud platform of the live-streaming app. An average of 1.91 (SD 0.32) videos and photographs of stay-at-home workouts and eye relaxation activities per intervention group student per day were uploaded to the live-streaming app (1.63, SD 0.31, during weekdays; 2.14, SD 0.80, on weekends). All students uploaded at least one video or photograph per day in the intervention group. The app includes the essential feature of a data-monitoring system, and detailed utilization data are presented in Figure 2.

The square-root–transformed self-reported anxiety score (main study outcome) fell by –0.23 (95% CI –0.27 to –0.20) in the intervention group and rose (worsened) by 0.12 (95% CI 0.09-0.16) in the controls by the end of the study (Table 1). The change in anxiety score was significantly greater in the intervention group compared to the controls (difference –0.36, 95% CI –0.63 to –0.08; *P*=.02). Significant associations were also found between changes in many SCAS subscale scores and the intervention (Supplementary Table S9, Multimedia Appendix 1). Change in self-reported eye strain was also significantly greater in the intervention vs the control group (intervention group: –0.08, 95% CI –0.10 to 0.06; control group: 0.07, 95% CI 0.05-0.09; difference –0.15, 95% CI –0.26 to –0.03; *P*=.02). The changes in sleep disturbance score (*P*=.23), screen time (*P*=.84), and reading time (*P*=.47) during the 2-week follow-up did not differ significantly between study groups (Table 1).

**Table 1:** Comparisons of change between study groups in self-reported anxiety, eye strain, sleep score, and time spent on different near-work activities between baseline and the 2-week follow-up (intention-to-treat analysis).

Variable			Intervention group (n=485), mean (95% CI)	Control group (n=469), mean (95% CI)	Difference in change between groups, mean (95% CI)	*P* value^a^
**Anxiety score^b,c^**						
	Baseline		3.72 (3.69 to 3.76)	3.67 (3.64 to 3.70)		
	2-week follow-up		3.49 (3.46 to 3.52)	3.79 (3.76 to 3.83)		
	Change (follow-up – baseline)		–0.23 (–0.27 to –0.20)	0.12 (0.09 to 0.16)	–0.36 (–0.63 to –0.08)	.02^d^
**Eye strain score^b,c^**						
	Baseline		1.21 (1.19 to 1.23)	1.08 (1.06 to 1.10)		
	2-week follow-up		1.13 (1.11 to 1.15)	1.15 (1.12 to 1.18)		
	Change (follow-up – baseline)		–0.08 (–0.10 to 0.06)	0.07 (0.05 to 0.09)	–0.15 (–0.26 to –0.03)	.02^d^
**Sleep disturbance score^b,c^**						
	Baseline		2.51 (2.50 to 2.52)	2.53 (2.53 to 2.54)		
	2-week follow-up		2.57 (2.56 to 2.58)	2.55 (2.54 to 2.56)		
	Change (follow-up – baseline)		0.06 (0.05 to 0.07)	0.01 (0.002 to 0.02)	0.05 (–0.03 to 0.13)	.22
**Average daily time spent in near work, hours**						
	**Screen time^b^**					
		Baseline	2.68 (2.67 to 2.69)	2.69 (2.68 to 2.70)		
		2-week follow-up	2.61 (2.60 to 2.62)	2.61 (2.60 to 2.63)		
		Change (follow-up – baseline)	–0.07 (–0.08 to –0.05)	–0.08 (–0.09 to –0.06)	0.01 (–0.10 to 0.12)	.84
	**Reading time^b^**					
		Baseline	1.37 (1.35 to 1.38)	1.29 (1.28 to 1.30)		
		2-week follow-up	1.34 (1.32 to 1.35)	1.21 (1.20 to 1.22)		
		Change (follow-up – baseline)	–0.03 (–0.05 to –0.01)	–0.08 (–0.09 to –0.06)	0.05 (–0.09 to 0.18)	.47

^a^Linear regression models adjusting for cluster effects within schools.

^b^Square root transformed.

^c^Higher score indicates greater severity.

^d^Significant.

In linear regression models, randomization to receive the peer-to-peer live-streaming intervention was associated with a significant reduction in self-reported anxiety compared to the controls (β=–0.36, 95% CI –0.63 to –0.08; *P*=.02), after adjusting for sex and household income (Supplementary Table S5, Multimedia Appendix 1). Results based on parentally reported anxiety scores were consistent (Supplementary Table S6, Multimedia Appendix 1).

## Discussion

### Principal Results

Our findings from a geographically representative region in urban China showed that a digital behavior change intervention effectively reduced anxiety and eye strain during children’s homeschooling, without increasing overall screen time. This digital tool might be useful during the COVID-19 pandemic, which has led to widespread school closures, with over a billion children around the world receiving online homeschooling, potentially increasing risk of anxiety and eye strain [10,30,31].

### Comparison With Prior Work

A recent review suggested that children’s mental and visual health may suffer during home confinement and online schooling [32]. These effects may result from reduced face-to-face social interaction, persistent and intense screen exposure, prolonged time spent on near-work activities, and the disruption of normal life rhythms, all of which may contribute to increased anxiety, eye strain, and sleep disturbance [33]. Children with preexisting mental health conditions such as depression may be especially susceptible to such problems [7].

The app only permitted users to post content related to exercise and activities promoting eye relaxation. This focus on activities highly relevant to both parents and children during the COVID-19 lockdown may explain the high study participation rate of over 95% and the large counts of children who shared photos or video content on a daily basis (Figure 2). We did not record any adverse events related to our intervention, nor did we observe any significant increase in screen time in the intervention group (Table 1). This latter finding is significant in view of concerns that children’s use of smartphones and social media may increase the risk of attention-deficit disorder, sleep disturbance, obesity, and other conditions [18,34,35]. Further studies are needed to investigate the long-term impacts of our digital intervention on children’s social media behavior.

### Strengths and Limitations

Strengths of the current study include the fact that participants were recruited from a population-representative sample of schools in a typical Chinese urban region, allowing our findings to be generalized to a wider range of Chinese children affected by the COVID-19 lockdown. Second, because of social media’s reliance on peer support, our protocol followed a cluster randomized design, which minimized the risk of cross-contamination in social media use between study groups. Thirdly, children in both study groups received the same online courses, ensuring intergroup homogeneity except with respect to the intervention. Finally, the results of primary, secondary, and sensitivity analyses were in full agreement; for example, there was concordance between self- and parentally reported anxiety scores (Supplementary Table S7, Multimedia Appendix 1), indicating the robustness and reliability of our findings.

Study limitations must also be acknowledged. First, masking of participants was not feasible due to the nature of the intervention, so the possibility of placebo effects cannot be excluded. However, good agreement between parental and self-report of anxiety makes this somewhat less likely. Second, data for the main outcome were obtained by questionnaires and were thus based on an evaluation of symptoms, rather than a clinical diagnosis of anxiety disorder. Regardless, the reproducibility of this questionnaire has been well validated [21], and, as mentioned above, parental and self-reported scores were in accord (Supplementary Table S7, Multimedia Appendix 1). Third, the study design allowed for only 2 weeks of follow-up. Previous studies have shown that interventions of this length can be effective [36-38], and such brief periods of engagement may be particularly applicable to unpredictable, short-term school closures [39], which may become more common in the future with additional waves of the COVID-19 pandemic. For example, a brief (2-3 week) lay counsellor–delivered, problem-solving intervention was found to be effective for adolescents with diverse mental health problems [38]. Nevertheless, it is certain that a longer follow-up will provide additional information on the robustness and stability of this intervention effect. Fourth, only Chinese children were included in the study, and the effectiveness of live-streaming interventions to reduce children’s anxiety and eye strain must be validated in other settings. Fifth, due to the nature of our behavior intervention, we could not exclude the possibility that the changes in children’s anxiety and eye strain may have been caused by other factors (eg, the increased interactions between children and parents), rather than a change in the behavior itself. In addition, the questionnaires were not fully validated for online use, which is the only means that can be used during COVID-19–related home learning. Finally, the urban sample used in the study may not be representative of China, not only because of the lack of smartphones in less developed parts of China but also due to the higher probability of separation between children and their parents due to labor migration. Our results therefore need to be interpreted with caution. 

### Conclusions

In summary, this study demonstrated that a novel digital behavior change intervention can significantly reduce anxiety and eye strain in homeschooled children. Further work is needed to determine whether these results can be extended to other aspects of pediatric and adult health, and to different geographic and cultural settings.

## Appendix

Multimedia Appendix 1 Supplementary content, tables, and figures.

Multimedia Appendix 2 CONSORT-eHEALTH checklist (V 1.6.1).

Multimedia Appendix 3 CHERRIES checklist.

## Abbreviations

CHERRIES: Checklist for Reporting Results of Internet E-Surveys
CONSORT: Consolidated Standards of Reporting Trials
CVS-Q: Computer Vision Syndrome Questionnaire
PROMIS: Patient-Reported Outcomes Measurement Information System
REAP: Recess and Exercise Advocacy Program
SCAS: Spence Children’s Anxiety Scale
SCAS-P: Spence Children’s Anxiety Scale for Parent
UNESCO: United Nations Educational, Scientific and Cultural Organization
