# Supplementary material for: A Peer-to-Peer Live-Streaming Intervention for Children During COVID-19 Homeschooling to Promote Physical Activity and Reduce Anxiety and Eye Strain: Cluster Randomized Controlled Trial
Source: J Med Internet Res. 2021 Apr 30;23(4):e24316. doi: 10.2196/24316 (PMC8092026; doi:10.2196/24316)
Supplement: Multimedia Appendix 1 [file jmir_v23i4e24316_app1.doc]

Supplementary Online Content, Tables, and Figures.

**A Peer-to-Peer Live Streaming Intervention for Children during COVID-19 Home Schooling to Promote Physical Activity and Reduce Anxiety and Eye Strain: Cluster Randomized Controlled Trial**

Table of Contents

**Supplementary** Figure **S**1. Geographic distribution of the middle schools included in this trial.

**Supplementary Table S1.** The number of students in each grade in the middle schools in Duanzhou district, Zhaoqing city, China.

**Supplementary Table S2.** Example of curriculum of the middle schools in Duanzhou district, Zhaoqing city, China.

**Supplementary Table S3.** Reasons for non-participation at baseline.

**Supplementary Table S4.** Baseline characteristics of the participants.

**Supplementary Table S5.** Intention to treat analysis for linear regression model of potential factors on the change of the self-report anxiety score.

**Supplementary Table S6.** Comparisons between study groups in parent-report anxiety, exercise time and eye relaxing time.

**Supplementary Table S7.** Correlation between child-reported anxiety score and parent-reported anxiety score.

**Supplementary Table S8.** Intention to treat analysis for linear regression model of potential factors on the change of the parent-report anxiety score.

**Supplementary Table S9.** Association between the intervention treatment and SCAS sub-scale scores.

**Supplementary Method S1.** Software development and technical considerations.

**Supplementary Method S2.** Questionnaires.

- **Questionnaire 2.1:** Spence Children's Anxiety Scale (SCAS) questionnaire (Child-report version).
- **Questionnaire 2.2:** Computer Vision Syndrome Questionnaire (CVS-Q).
- **Questionnaire 2.3:** PROMIS pediatric sleep disturbance questionnaire.
- **Questionnaire 2.4:** Children’s near work activities questionnaire.
- **Questionnaire 2.5:** Spence Children's Anxiety Scale (SCAS) for Parent questionnaire (Parent-report version).

**Supplementary Method S3.** Research protocol and data analysis plan as submitted in our Institutional Review Board application

**Supplementary** Figure **S**1. Geographic distribution of the included middle schools.

Zhaoqing is one of the urban cities in Guangdong Province in China. Duanzhou district is the political and economic center of Zhaoqing. This district occupies an area of 152.3 square kilometers, with a population of 480 thousand people (2017 Census). A total of 12 middle school (Red bubbles) in Duanzhou District were included in the study.


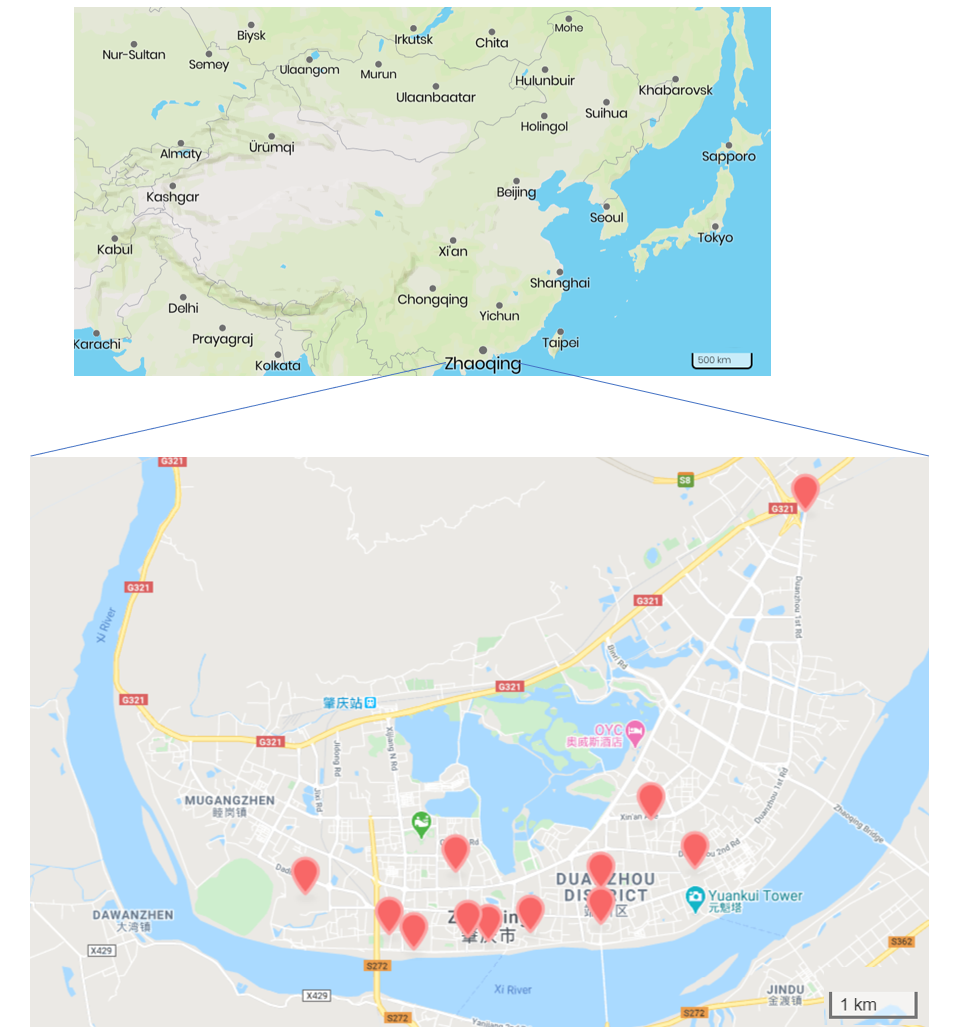


**Supplementary Table S1.** The number of students in each grade in the middle schools in Duanzhou district, Zhaoqing city, China.

| **No.**^a^ | **School** | **7^th^ grade** | **8^th^ grade** | **9^th^ grade** | **All** |
| --- | --- | --- | --- | --- | --- |
| 1 | Songde Middle School | 449 | 433 | 514 | 1396 |
| 2 | Railway Middle School | 76 | 80 | 80 | 236 |
| 3 | Middle school affiliated Zhaoqing college | 292 | 232 | 235 | 759 |
| 4 | The 4^th^ Zhaoqing Middle School | 544 | 543 | 523 | 1610 |
| 5 | The 12^th^ Zhaoqing Middle School | 284 | 299 | 350 | 933 |
| 6 | The 6^th^ Zhaoqing Middle School | 518 | 503 | 663 | 1684 |
| 7 | The 8^th^ Zhaoqing Middle School | 246 | 235 | 224 | 705 |
| 8 | The 5^th^ Zhaoqing Middle School | 399 | 380 | 403 | 1182 |
| 9 | The 2^nd^ Zhaoqing Middle School | 373 | 392 | 433 | 1198 |
| 10 | The 1^st^ Zhaoqing Middle School | 696 | 650 | 652 | 1998 |
| 11 | Duanzhou Middle School | 296 | 190 | 216 | 702 |
| 12 | Dalong Middle School | 33 | 20 | 28 | 81 |
| 13 | Geological Middle School | 557 | 547 | 554 | 1658 |
| 14 | Jiamei Middle School | 176 | 139 | 135 | 450 |

^a^A total of 14 middle schools in this area. Two school (School 2 and School 12) were ineligible because the number of grade 7 students was less than the subjects required per cluster.

**Supplementary Table S2.** Example of Curriculum of the Middle School.

| Time/Subject | | Monday | Tuesday | Wednesday | Thursday | Friday |
| --- | --- | --- | --- | --- | --- | --- |
| AM | First class  9:00-9:20 | Chinese | Mathematics | English | Chinese | Mathematics |
|  | **Class recess**  **9:20-9:30** |  | | | | |
|  | Second class  9:30-9:50 | Morality & Law | History | Geography | Biology | Morality & Law |
|  | **Class recess**  **9:50-10:10** |  | | | | |
|  | Third class  10:10-10:30 | English | Chinese | Mathematics | English | History |
|  | **Class recess**  **10:30-10:40** |  | | | | |
|  | Fourth class  9:00-9:20 | Class meeting | Music | Art | Information technology | Calligraphy |
| Noon rest period | | | | | | |
| PM | Fifth class  15:00-15:20 | Sports & Health | Mental health education | Sports & Health | Labor & Technique | Sports & Health |
|  | **Class recess**  **15:20-15:40** |  | | | | |
|  | Sixth class  15:40-16:00 | Provincial Course | Provincial Course | Provincial Course | Provincial Course | Provincial Course |

**Supplementary Table S3. Reasons for non-participation at baseline.**

| **Reasons** | **All** | **Intervention group** | **Control group** |
| --- | --- | --- | --- |
| Unsuitable | 17 (30.9%) | 9 (36.0%) | 8 (26.7%) |
| Decline | 10 (18.2%) | 4 (16.0%) | 6 (20.0%) |
| Unavailable or unreachable | 28 (50.9%) | 12 (48.0%) | 16 (53.3%) |
| All | 55 (100%) | 25 (100%) | 30 (100%) |

**Supplementary Table S4.** Baseline characteristics of the participants.

|  | | **Intervention group (n = 485)** | **Control group (n = 469)** |
| --- | --- | --- | --- |
| Age, mean (SD), year | | 13.5 (0.50) | 13.5 (0.50) |
| Boys, n (%) | | 248 (51.1%) | 251 (53.5%) |
| Wearing glasses, n (%) | | 242 (49.9%) | 240 (51.2%) |
| Household income, US$/month | |  |  |
|  | Less than 707 | 168 (34.6%) | 150 (32.0%) |
|  | 707 – 1,414 | 143 (29.5%) | 154 (32.8%) |
|  | More than 1,414 | 174 (35.9%) | 165 (35.2%) |
| Either parent currently smokes, n (%) | | 245 (50.5%) | 227 (48.4%) |
| Highest level of parental education attained, n (%) | |  |  |
|  | Primary school or less | 182 (37.5%) | 151 (32.2%) |
|  | Secondary school | 136 (28.0%) | 142 (30.3%) |
|  | Bachelor’s degree or above | 167 (34.5%) | 176 (37.5%) |

Data are presented as mean (standard deviation) or number (%).

**Supplementary Table S5.** Intention to treat analysis for linear regression model of potential factors on the change of the self-report anxiety score^a^.

| **Variable** | **Univariable regression (n = 954)**^b^ | |  | **Multivariable regression (n = 954)**^b^ | |
| --- | --- | --- | --- | --- | --- |
|  | **β (95% CI)** | ***P* value** |  | **β (95% CI)** | ***P* value** |
| Intervention group | -0.36 (-0.63, -0.08) | **.02** |  | -0.36 (-0.64, -0.08) | **.02** |
| Age | -0.03 (-0.36, 0.29) | .83 |  | - | - |
| Boys (Girls as reference) | 0.25 (-0.04, 0.54) | .08 |  | 0.23 (-0.05, 0.52) | .10 |
| Wearing glasses vs. No wearing glasses | 0.05 (-0.20, 0.30) | .68 |  | - | - |
| Household income, US$/month |  |  |  |  |  |
| Less than 707 | Ref | - |  | Ref | - |
| 707 - 1,414 | -0.32 (-0.64, -0.001) | **.05** |  | -0.33 (-0.67, 0.003) | .05 |
| More than 1,414 | -0.02 (-0.35, 0.30) | .88 |  | -0.03 (-0.36, 0.30) | .84 |
| Parental current smoking status vs. No smoking | 0.05 (-0.23, 0.34) | .69 |  | - | - |
| Parental education attainment |  |  |  |  |  |
| Primary school or less | Ref | - |  | - | - |
| Secondary school | -0.11 (-0.42, 0.21) | .48 |  | - | - |
| Bachelor degree or over | -0.12 (-0.35, 0.12) | .30 |  | - | - |

β = Coefficient parameter estimate, CI = Confidence interval

^a^ Square root transformed

^b^ Variables with p value < .20 in the univariable regression analysis were included in the multivariable linear regression model. Linear regression models were adjusted for cluster effects. Multiple imputation was performed for missing data.

**Supplementary Table S6.** Comparisons between study groups in parent-report anxiety, exercise time and eye relaxing time.

|  | **Intervention group (n=485)** | **Control group (n=469)** | ***P* value**^c^ |
| --- | --- | --- | --- |
| Parent-report anxiety score, mean (95% confidence interval)^ab^ |  |  |  |
| Baseline | 4.70 (4.67, 4.72) | 4.60 (4.58, 4.63) |  |
| 2-week follow-up | 4.20 (4.18, 4.23) | 4.40 (4.37, 4.43) |  |
| Change (Follow-up – Baseline) | -0.49 (-0.52, -0.46) | -0.20 (-0.23, -0.17) | **.04** |
| Average minutes of exercise during class break in study period, mean (95% confidence interval)^a^ | 1.51 (1.35, 1.67) | 1.23 (1.10, 1.36) | **.03** |
| Average minutes of eye relaxing during class break in study period, mean (95% confidence interval)^a^ | 0.28 (0.23, 0.33) | 0.21 (0.18, 0.25) | .13 |

^a^ Square root transformed

^b^ Higher score indicate more severity.

^c^ T-test adjusting for cluster effects.

**Supplementary Table S7.** Correlation between child-reported anxiety score and parent-reported anxiety score.

| **Parameter** | **Pearson correlation** | **ICC** |
| --- | --- | --- |
| Anxiety score at baseline^a^ | 0.596^b^ | 0.716 |
| Anxiety score at 2 weeks follow-up^a^ | 0.614^b^ | 0.753 |

ICC=Intraclass Correlations

^a^ Square root transformed

^b^ *P* < .001

**Supplementary Table S8.** Intention to treat analysis for linear regression model of potential factors on the change of the parent-report anxiety score^a^.

| **Variable** | **Univariable regression (n = 954)**^b^ | |  | **Multivariable regression (n = 954)**^b^ | |
| --- | --- | --- | --- | --- | --- |
|  | **β (95% CI)** | ***P* value** |  | **β (95% CI)** | ***P* value** |
| Intervention group | -0.29 (-0.56, -0.02) | **.04** |  | -0.31 (-0.58, -0.04) | **.03** |
| Age | 0.08 (-0.19, 0.35) | .51 |  | - | - |
| Boys (Girls as reference) | 0.08 (-0.18, 0.33) | .52 |  |  |  |
| Wearing glasses vs. No wearing glasses | -0.04 (-0.29, 0.22) | .77 |  | - | - |
| Household income, US$/month |  |  |  |  |  |
| Less than 707 | Ref | - |  | Ref | - |
| 707 - 1414 | -0.14 (-0.35, 0.08) | .19 |  | -0.14 (-0.36, 0.08) | .19 |
| More than 1414 | -0.01 (-0.29, 0.28) | .96 |  | 0.01 (-0.27, 0.28) | .96 |
| Parental current smoking vs. No smoking | 0.08 (-0.13, 0.29) | .42 |  | - | - |
| Parental education attainment |  |  |  |  |  |
| Primary school or less | Ref | - |  | Ref |  |
| Secondary school | -0.22 (-0.52, 0.07) | .13 |  | -0.23 (-0.53, 0.07) | .11 |
| Bachelor degree or over | -0.26 (-0.56, 0.04) | .09 |  | -0.28 (-0.57, 0.01) | .06 |

β = Coefficient parameter estimate, CI = Confidence interval

^a^ Square root transformed

^b^ Variables with *P* value < .20 in the univariable regression analysis were included in the multivariable linear regression model. Linear regression models were adjusted for cluster effects. Multiple imputation was performed for missing data.

**Supplementary Table S9. Association between the intervention treatment and changes in SCAS sub-scale scores**.

|  | **Panic/agoraphobia** ^a^ | |  | **Separation anxiety** ^a^ | |  | **Social phobia** ^a^ | |
| --- | --- | --- | --- | --- | --- | --- | --- | --- |
|  | **β (95% CI)** | ***P* value** |  | **β (95% CI)** | ***P* value** |  | **β (95% CI)** | ***P* value** |
| Intervention | -0.62 (-1.09, -0.16) | **.008** |  | -0.32 (-0.67, 0.02) | .07 |  | -0.68 (-1.21, -0.16) | **.01** |
| Control | Ref |  |  | Ref |  |  | Ref |  |
|  |  |  |  |  |  |  |  |  |
|  | **Fears of physical injury** ^a^ | |  | **Obsessions/compulsions** ^a^ | |  | **Generalized anxiety** ^a^ | |
|  | **β (95% CI)** | ***P* value** |  | **β (95% CI)** | ***P* value** |  | **β (95% CI)** | ***P* value** |
| Intervention | -0.69 (-1.08, -0.29) | **.001** |  | -0.45 (-0.91, 0.01) | .06 |  | -0.66 (-1.04, -0.27) | **.001** |
| Control | Ref |  |  | Ref |  |  | Ref |  |

SCAS = Spence Children's Anxiety Scale, β = Coefficient parameter estimate, CI = Confidence interval

^a^ Multivariable linear regression model after adjusting for variables including household income and parental education level.

**Supplementary Method S1.** Live streaming application development and technical consideration.

Long-term exposure to screen may cause eye discomfort such as eye strain. The study’s software engineer developed a live-streaming application (App) that offers Recess and Exercise Advocacy Program (REAP), allowing users to shoot short videos or photos related to their physical exercise or eye relaxation (e.g., staring out of the window) using their smartphones. The REAP is a mini program that integrate directly into WeChat and runs in the WeChat application. This is different from traditional apps in that it requires no download and no install.

The REAP encourage students to take different forms of physical relaxation exercises, take a break between classes, look out the window and look into the distance, so that they could allow their eyes to rest and relax during class recesses at home. With the help of their parents or by making selfies, children could livestream their own workout videos/photos and share them to the live-streaming platform. After uploading, children share motivational messages or hit the like button to increase their engagement with the program. The trial coworkers (class administrators) who are not involved in this study supervise and approve all the uploaded contents.

**Recess and Exercise Advocacy Program (REAP)**
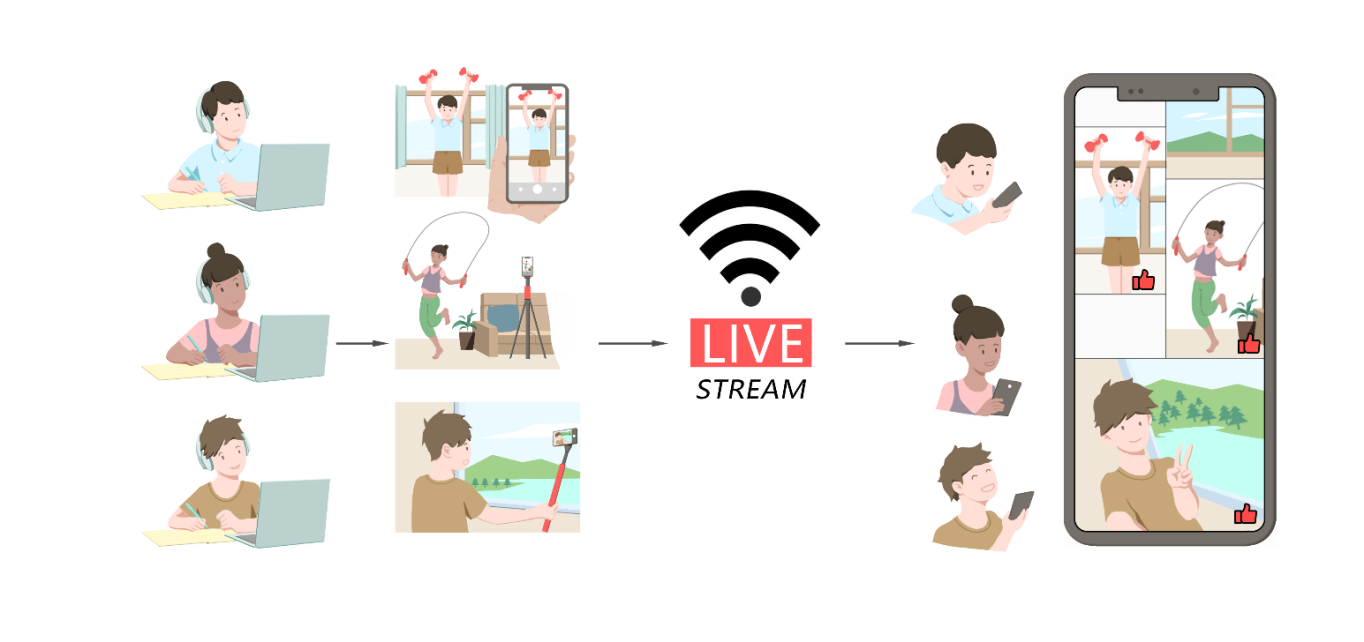


**(1) Schematic diagram of the process of live streaming application.**


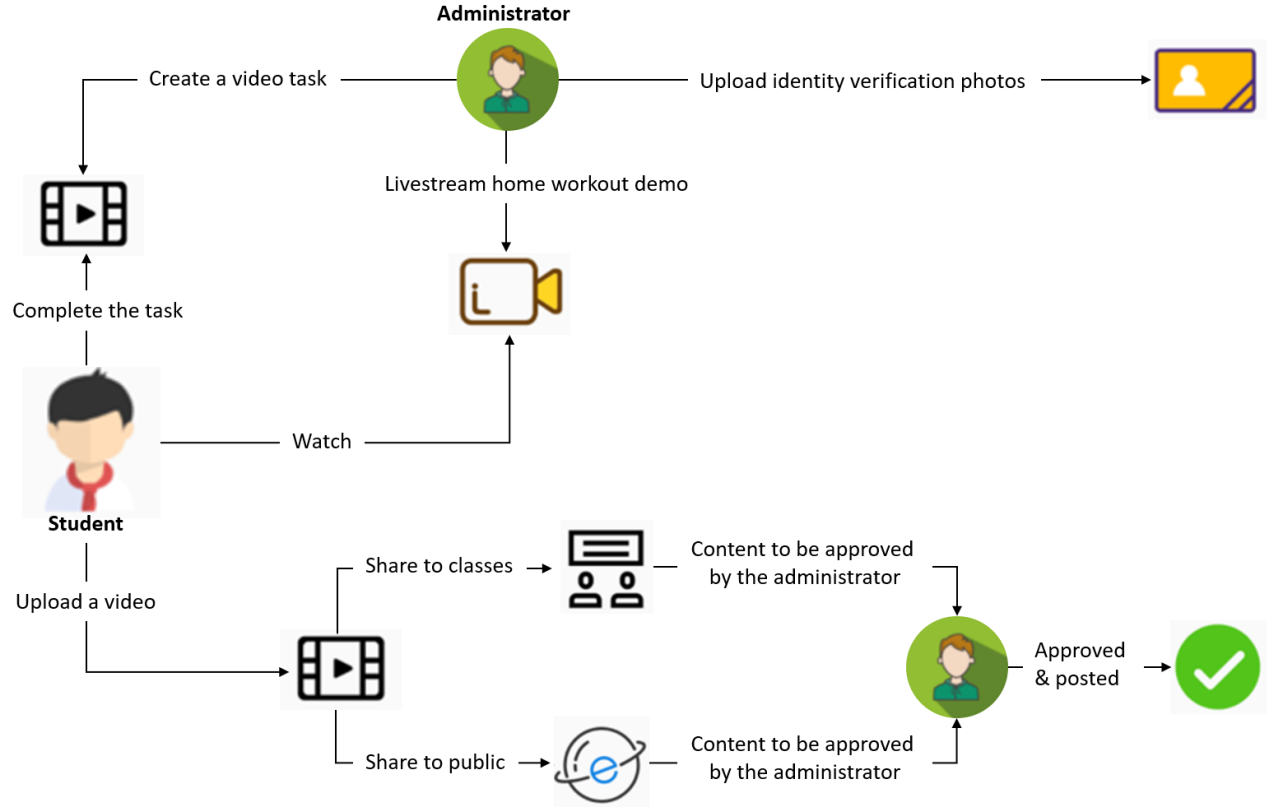


**(2) Tutorial for Master Administrators.**

The Master Administrator has the sole responsibility for approving request for the Class Administrator access. Class administrator roles should be approved by the Master Administrator.


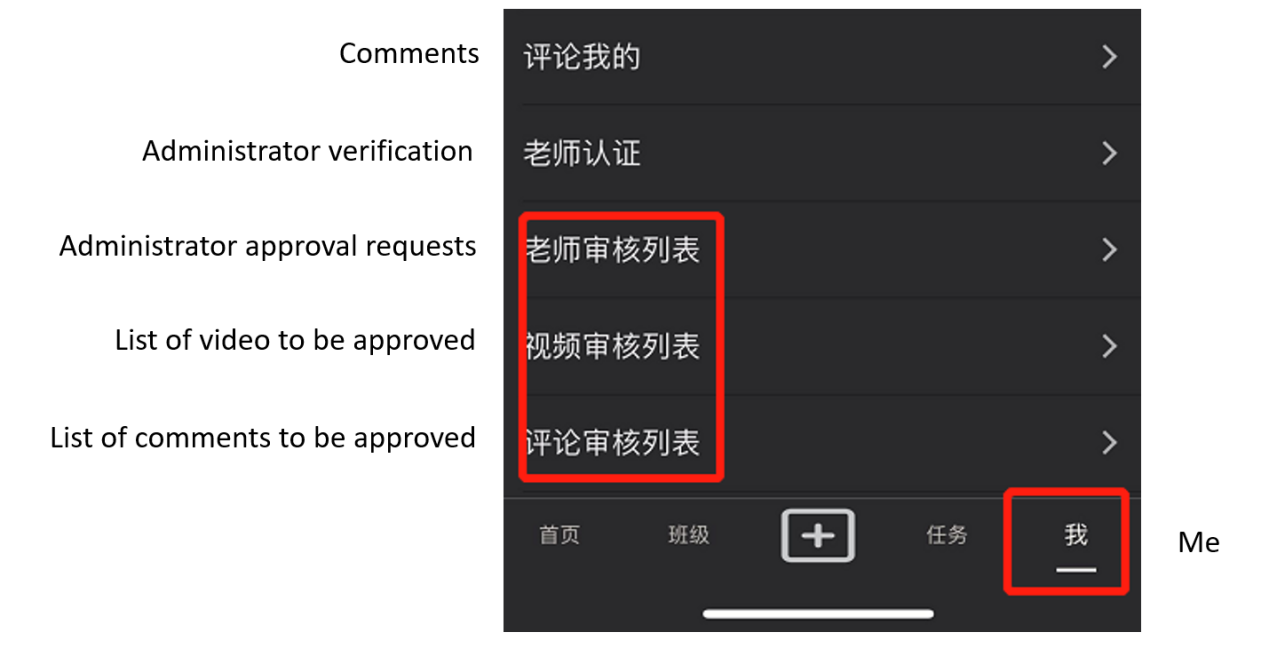


**(3) Tutorial for Class Administrators**

3.1 Class administrators are responsible for creation of work out demo videos (by clicking the "+" button), and edit, approve or remove video.


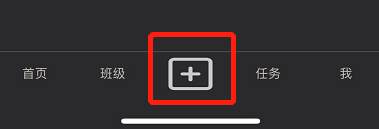


3.2 Post demo video


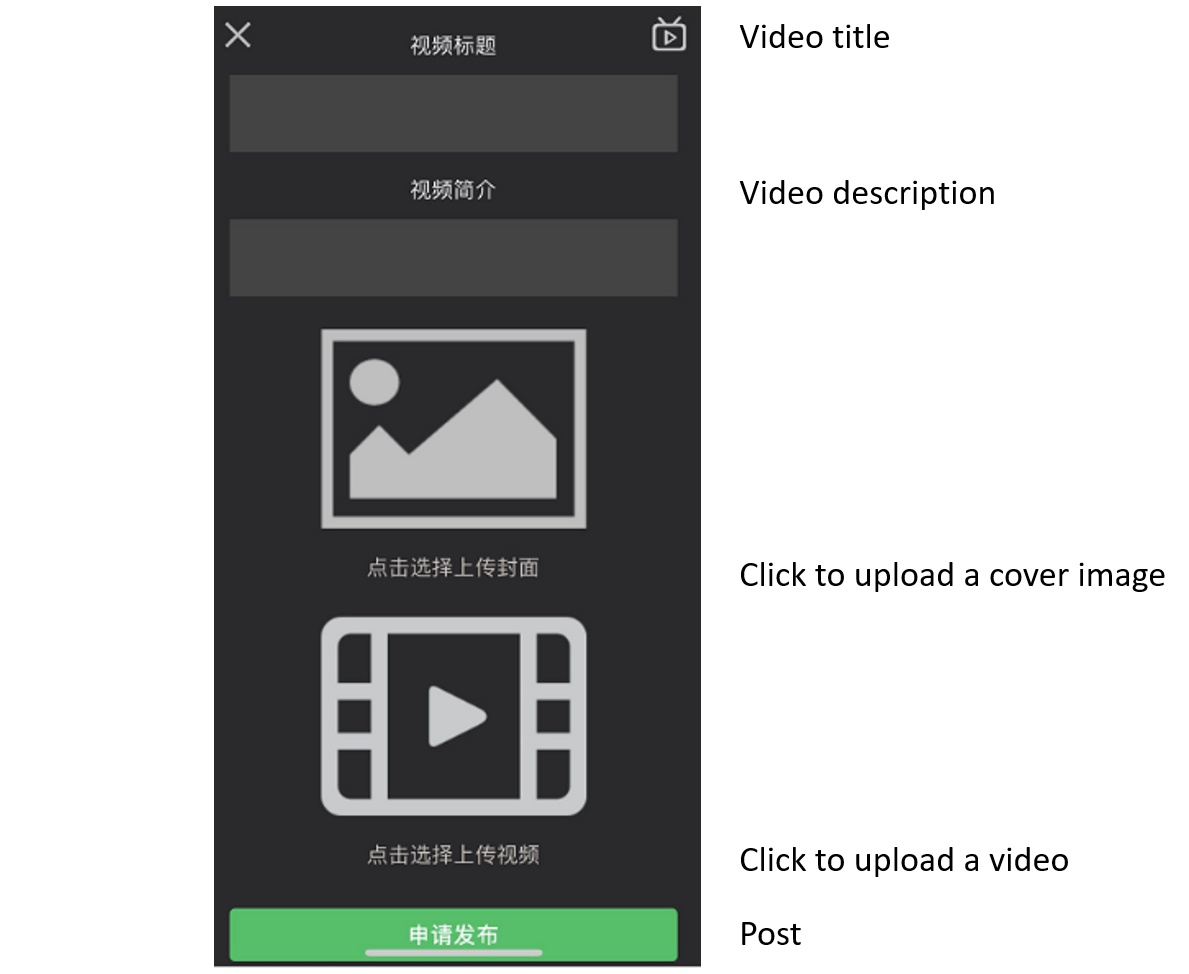


3.3 Live broadcast


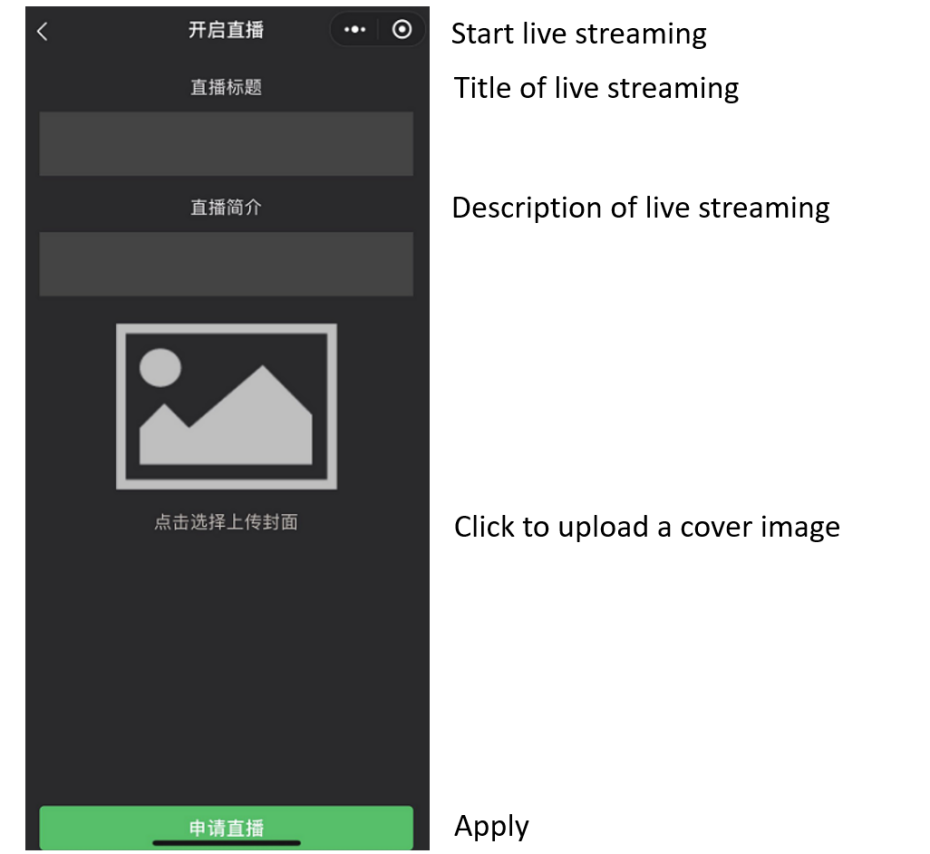


**(4) Tutorial for students**

REAP is a student-driven digital portfolio that encourages students to broadcast home workout and relaxation videos publicly or just to classmates. After the broadcast, students can post the video to the home page and download the video for archival purposes.

4.1 Post video


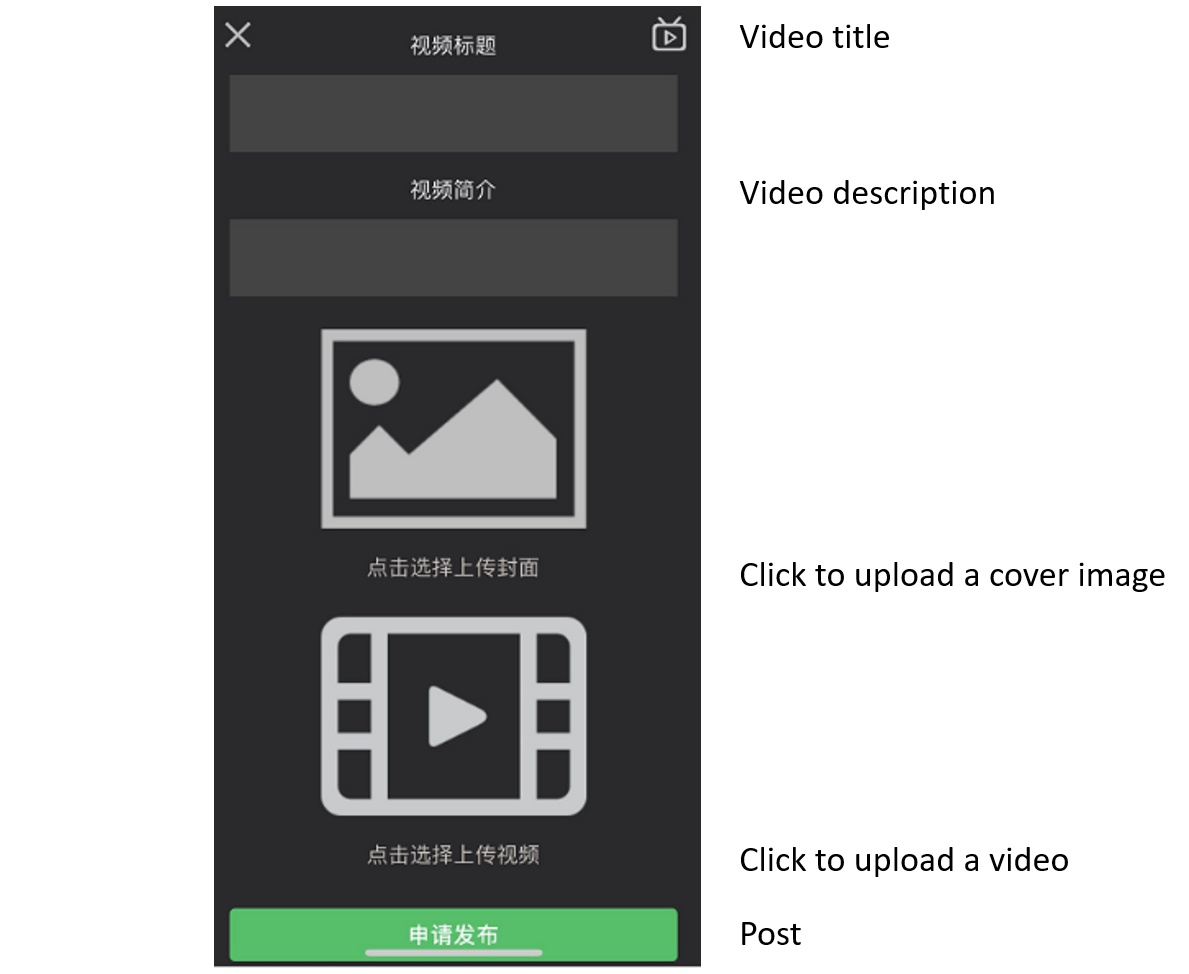


**(5) How to assess the REAP live streaming application.**

Login the Wechat system and scan the QR code below

**
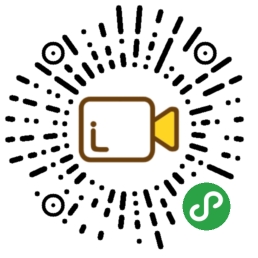
**

**Supplementary Method S2.** Questionnaire.

**Questionnaire 2.1. Spence Children’s Anxiety Scale (child-report version).**

| **Question** | **Details** |
| --- | --- |
| I worry about things | one of {Never, Sometimes, Often, Always} |
| I am scared of the dark | one of {Never, Sometimes, Often, Always} |
| When I have a problem, I get a funny feeling in my stomach | one of {Never, Sometimes, Often, Always} |
| I feel afraid | one of {Never, Sometimes, Often, Always} |
| I would feel afraid of being on my own at home | one of {Never, Sometimes, Often, Always} |
| I feel scared when I have to take a test | one of {Never, Sometimes, Often, Always} |
| I feel afraid if I have to use public toilets or bathrooms | one of {Never, Sometimes, Often, Always} |
| I worry about being away from my parents | one of {Never, Sometimes, Often, Always} |
| I feel afraid that I will make a fool of myself in front of people | one of {Never, Sometimes, Often, Always} |
| I worry that I will do badly at my school work | one of {Never, Sometimes, Often, Always} |
| I am popular amongst other kids my own age | one of {Never, Sometimes, Often, Always} |
| I worry that something awful will happen to someone in my family | one of {Never, Sometimes, Often, Always} |
| I suddenly feel as if I can’t breathe when there is no reason for this | one of {Never, Sometimes, Often, Always} |
| I have to keep checking that I have done things right (like the switch is off, or the door is locked) | one of {Never, Sometimes, Often, Always} |
| I feel scared if I have to sleep on my own | one of {Never, Sometimes, Often, Always} |
| I have trouble going to school in the mornings because I feel nervous or afraid | one of {Never, Sometimes, Often, Always} |
| I am good at sports | one of {Never, Sometimes, Often, Always} |
| I am scared of dogs | one of {Never, Sometimes, Often, Always} |
| I can’t seem to get bad or silly thoughts out of my head | one of {Never, Sometimes, Often, Always} |
| When I have a problem, my heart beats really fast | one of {Never, Sometimes, Often, Always} |
| I suddenly start to tremble or shake when there is no reason for this | one of {Never, Sometimes, Often, Always} |
| I worry that something bad will happen to me | one of {Never, Sometimes, Often, Always} |
| I am scared of going to the doctors or dentists | one of {Never, Sometimes, Often, Always} |
| When I have a problem, I feel shaky | one of {Never, Sometimes, Often, Always} |
| I am scared of being in high places or lifts (elevators) | one of {Never, Sometimes, Often, Always} |
| I am a good person | one of {Never, Sometimes, Often, Always} |
| I have to think of special thoughts to stop bad things from happening (like numbers or words) | one of {Never, Sometimes, Often, Always} |
| I feel scared if I have to travel in the car, or on a Bus or a train | one of {Never, Sometimes, Often, Always} |
| I worry what other people think of me | one of {Never, Sometimes, Often, Always} |
| I am afraid of being in crowded places (like shopping centres, the movies, buses, busy playgrounds) | one of {Never, Sometimes, Often, Always} |
| I feel happy | one of {Never, Sometimes, Often, Always} |
| All of a sudden, I feel really scared for no reason at all | one of {Never, Sometimes, Often, Always} |
| I am scared of insects or spiders | one of {Never, Sometimes, Often, Always} |
| I suddenly become dizzy or faint when there is no reason for this | one of {Never, Sometimes, Often, Always} |
| I feel afraid if I have to talk in front of my class | one of {Never, Sometimes, Often, Always} |
| My heart suddenly starts to beat too quickly for no reason | one of {Never, Sometimes, Often, Always} |
| I worry that I will suddenly get a scared feeling when there is nothing to be afraid of | one of {Never, Sometimes, Often, Always} |
| I like myself | one of {Never, Sometimes, Often, Always} |
| I am afraid of being in small closed places, like tunnels or small rooms. | one of {Never, Sometimes, Often, Always} |
| I have to do some things over and over again (like washing my hands, cleaning or putting things in a certain order) | one of {Never, Sometimes, Often, Always} |
| I get bothered by bad or silly thoughts or pictures in my mind | one of {Never, Sometimes, Often, Always} |
| I have to do some things in just the right way to stop bad things happening | one of {Never, Sometimes, Often, Always} |
| I am proud of my school work | one of {Never, Sometimes, Often, Always} |
| I would feel scared if I had to stay away from home overnight | one of {Never, Sometimes, Often, Always} |
| Is there something else that you are really afraid of? | one of {Never, Sometimes, Often, Always} |

**Questionnaire 2.2.** Computer Vision Syndrome Questionnaire (CVS-Q).

| **Question** | **Details** | |
| --- | --- | --- |
|  | **a. Frequency** | **b. Intensity** |
| Burning | one of {Never, Occasionally, Often or always} | one of {Moderate, Intense} |
| Itching | one of {Never, Occasionally, Often or always} | one of {Moderate, Intense} |
| Feeling of a foreign body | one of {Never, Occasionally, Often or always} | one of {Moderate, Intense} |
| Tearing | one of {Never, Occasionally, Often or always} | one of {Moderate, Intense} |
| Excessive blinking | one of {Never, Occasionally, Often or always} | one of {Moderate, Intense} |
| Eye redness | one of {Never, Occasionally, Often or always} | one of {Moderate, Intense} |
| Eye pain | one of {Never, Occasionally, Often or always} | one of {Moderate, Intense} |
| Heavy eyelids | one of {Never, Occasionally, Often or always} | one of {Moderate, Intense} |
| Dryness | one of {Never, Occasionally, Often or always} | one of {Moderate, Intense} |
| Blurred vision | one of {Never, Occasionally, Often or always} | one of {Moderate, Intense} |
| Double vision | one of {Never, Occasionally, Often or always} | one of {Moderate, Intense} |
| Difficulty focusing for near vision | one of {Never, Occasionally, Often or always} | one of {Moderate, Intense} |
| Increased sensitivity to light | one of {Never, Occasionally, Often or always} | one of {Moderate, Intense} |
| Coloured halos around objects | one of {Never, Occasionally, Often or always} | one of {Moderate, Intense} |
| Feeling that sight is worsening | one of {Never, Occasionally, Often or always} | one of {Moderate, Intense} |
| Headache | one of {Never, Occasionally, Often or always} | one of {Moderate, Intense} |

**Questionnaire 2.3. PROMIS Sleep Disturbance Questionnaire (Short Form).**

| **Question** | **Details** |
| --- | --- |
| I had difficulty falling asleep in the past 7 days | one of {Not at all, A little bit, Somewhat, Quite a bit, Very much} |
| I was satisfied with my sleep in the past 7 days | one of {Not at all, A little bit, Somewhat, Quite a bit, Very much} |
| I slept through the night in the past 7 days | one of {Not at all, A little bit, Somewhat, Quite a bit, Very much} |
| I had a problem with my sleep in the past 7 days | one of {Not at all, A little bit, Somewhat, Quite a bit, Very much} |

**Questionnaire 2.4. Children’s Near Work Activities Questionnaire.**

| **Question** | **Detail** |
| --- | --- |
| **Screen time** |  |
| What is the average time for using smart-phone per day? | text input |
| What is the average time for watching TV per day? | text input |
| What is the average time for using computer per day? | text input |
| What is the average time for playing video game per day? | text input |
| **Reading time** |  |
| How many times of reading per week? | text input |
| What is the average time for reading per time? | text input |

**Questionnaire 2.5: Spence Children's Anxiety Scale (SCAS) questionnaire (Parent-report version).**

| **Question** | **Details** |
| --- | --- |
| My child worries about things | one of {Never, Sometimes, Often, Always} |
| My child is scared of the dark | one of {Never, Sometimes, Often, Always} |
| When my child has a problem, s(he) complains of having a funny feeling in his / her stomach | one of {Never, Sometimes, Often, Always} |
| My child complains of feeling afraid | one of {Never, Sometimes, Often, Always} |
| My child would feel afraid of being on his/her own at home | one of {Never, Sometimes, Often, Always} |
| My child is scared when s(he) has to take a test | one of {Never, Sometimes, Often, Always} |
| My child is afraid when (s)he has to use public toilets or bathrooms | one of {Never, Sometimes, Often, Always} |
| My child worries about being away from us / me | one of {Never, Sometimes, Often, Always} |
| My child feels afraid that (s)he will make a fool of him/herself in front of people | one of {Never, Sometimes, Often, Always} |
| My child worries that (s)he will do badly at school | one of {Never, Sometimes, Often, Always} |
| My child worries that something awful will happen to someone in our family | one of {Never, Sometimes, Often, Always} |
| My child complains of suddenly feeling as if (s)he can't breathe when there is no reason for this | one of {Never, Sometimes, Often, Always} |
| My child has to keep checking that (s)he has done things right (like the switch is off, or the door is locked) | one of {Never, Sometimes, Often, Always} |
| My child is scared if (s)he has to sleep on his/her own | one of {Never, Sometimes, Often, Always} |
| My child has trouble going to school in the mornings because (s)he feels nervous or afraid | one of {Never, Sometimes, Often, Always} |
| My child is scared of dogs | one of {Never, Sometimes, Often, Always} |
| My child can't seem to get bad or silly thoughts out of his / her head | one of {Never, Sometimes, Often, Always} |
| When my child has a problem, s(he) complains of his/her heart beating really fast | one of {Never, Sometimes, Often, Always} |
| My child suddenly starts to tremble or shake when there is no reason for this | one of {Never, Sometimes, Often, Always} |
| My child worries that something bad will happen to him/her | one of {Never, Sometimes, Often, Always} |
| My child is scared of going to the doctor or dentist | one of {Never, Sometimes, Often, Always} |
| When my child has a problem, (s)he feels shaky | one of {Never, Sometimes, Often, Always} |
| My child is scared of heights (eg being at the top of a cliff) | one of {Never, Sometimes, Often, Always} |
| My child has to think special thoughts (like numbers or words) to stop bad things from happening | one of {Never, Sometimes, Often, Always} |
| My child feels scared if (s)he has to travel in the car, or on a bus or train | one of {Never, Sometimes, Often, Always} |
| My child worries what other people think of him/her | one of {Never, Sometimes, Often, Always} |
| My child is afraid of being in crowded places (like shopping centers, the movies, buses, busy playgrounds) | one of {Never, Sometimes, Often, Always} |
| All of a sudden, my child feels really scared for no reason at all | one of {Never, Sometimes, Often, Always} |
| My child is scared of insects or spiders | one of {Never, Sometimes, Often, Always} |
| My child complains of suddenly becoming dizzy or faint when there is no reason for this | one of {Never, Sometimes, Often, Always} |
| My child feels afraid when (s)he has to talk in front of the class | one of {Never, Sometimes, Often, Always} |
| My child’s complains of his / her heart suddenly starting to beat too quickly for no reason | one of {Never, Sometimes, Often, Always} |
| My child worries that (s)he will suddenly get a scared feeling when there is nothing to be afraid of | one of {Never, Sometimes, Often, Always} |
| My child is afraid of being in small closed places, like tunnels or small rooms | one of {Never, Sometimes, Often, Always} |
| My child has to do some things over and over again (like washing his / her hands, cleaning or putting things in a certain order) | one of {Never, Sometimes, Often, Always} |
| My child gets bothered by bad or silly thoughts or pictures in his/her head | one of {Never, Sometimes, Often, Always} |
| My child has to do certain things in just the right way to stop bad things from happening | one of {Never, Sometimes, Often, Always} |
| My child would feel scared if (s)he had to stay away from home overnight | one of {Never, Sometimes, Often, Always} |
| Is there anything else that your child is really afraid of? | one of {Yes, No} |
| Please write down what it is, and fill out how often (s)he is afraid of this thing: | text input, one of {Never, Sometimes, Often, Always} |

**Supplementary Method S3. Research protocol and data analysis plan as submitted in our Institutional Review Board application**

**Unique Protocol ID:** 2020KYPJ045

**Brief Title:** Reducing Eye Strain and Anxiety Using a Digital Intervention During Online Learning Class Recess Among Children at Home: A Randomized Controlled Trial (RESILIENT)

**Official Title:** Reducing Eye Strain and Anxiety Using a Digital Intervention During Online Learning Class Recess Among Children at Home: A Randomized Controlled Trial

***Brief Summary of Research:***

In response to the coronavirus disease 2019 (COVID-19), many countries and regions have taken the decision of school closure after the confirmation of coronavirus cases in the general population.^1, 2^ In China, the burden of COVID-19 is among the highest in the world, with more than 80,000 cases confirmed by 11th March 2020. An estimate of 220 million children and adolescents are confined at home for weeks with inadequate level of physical activity and more susceptible to anxiety.^3^ There is also a concern of digital eye strain and myopia progression for many children who spend hours daily in front of a computer screen for recreation or learning purposes.^4-7^ Recess represents an essential scheduled period in a school day for psychological and physical relaxation.^8^ During the unusual period of home confinement, many schools have already issued physical activity requirement and policy recommending recess and physical activity breaks. However, recess could be easily skipped by students during online learning at home. Mobile health intervention offers a potential opportunity for capitalizing on digital technology as a feasible modality to encourage recess activities, especially in regions where social distancing is implemented as an emergency measure.^9^

In light of this gap, we have developed a novel peer-to-peer live-streaming application called the Recess and Exercise Advocacy Program (REAP), designed to encourage regular physical activity and relaxation of accommodation (near focusing) during online school recess periods. The aim of this study is to evaluate the effectiveness of this digital intervention in reducing anxiety syndrome (main outcome) and eye strain, compared to a conventional educational intervention among Chinese children during a period of home confinement occasioned by the COVID-19 pandemic.

To minimize the possibility of cross arm communication and contamination, we will conduct a cluster randomized controlled trial, with schools randomly assigned to either the intervention or control group. This would also allow for peer-to-peer support in the use of live-streaming application.

***Specific Aims (SA):***

Specific Aim 1 (SA1): To determine the effectiveness of REAP in relieving anxiety syndrome in children during home confinement and schooling.

Specific Aim 2 (SA2): To assess the effectiveness of REAP in relieving eye strain in children during home confinement and schooling.

Specific Aim 3 (SA3): To determine the impact of REAP on children’s sleep quality during home confinement and schooling.

Specific Aim 4 (SA4): To evaluate the impact of REAP on time spent on near work activities (i.e. reading, writing, computer/PAD use, smart phone, and watching TV).

***Eligibility of participants:***

Inclusion criteria:

1. Grade 7 (12-13 years old) students in Duanzhou District, Zhaoqing city;
2. Students under home confinement and enrolled in online learning courses, during the COVID-19 outbreak.

Exclusion criteria:

1. Autism spectrum disorders and pervasive developmental delay or disorder;
2. Mental retardation;
3. Psychotic disorders and schizophrenia;
4. Mania or hypomania disorders;
5. Suicidal behavior and/or acute plan that require higher level of care;
6. Participation in psychotherapy.

***Outcome Measures (OM):***

OM 1: Change in self-report anxiety score (primary outcome measure)

Change in anxiety will be measured by the Spence Children's Anxiety Scale (SCAS) designed by Spence (1998).^10^ The SCAS (45-item) is self-report scale with a 4-point Likert type, consisting of 44 items and one open-ended question. The reliability and validity of this questionnaire have been evaluated in Chinese children and adolescents.^11^

[Time Frame: baseline & at 2 weeks]

OM 2: Change in syndromes of eye strain

Syndromes of digital eye strain will be measured with the Computer Vision Syndrome Questionnaire (CVS-Q) designed by Seguí et al (2015).^12^ The self-reported CVS-Q questionnaire (16-item) evaluates the frequency (never, occasionally or often/always) and the intensity (moderate or intense) of 16 symptoms: burning, itching, feeling of a foreign body, tearing, excessive blinking, eye redness, eye pain, heavy eyelids, dryness, blurred vision, double vision, difficulty focusing for near vision, increased sensitivity to light, colored halos around objects, feeling that sight is worsening, and headache.

[Time Frame: baseline & at 2 weeks]

OM 3: Change in sleeping quality

The PROMIS pediatric sleep disturbance questionnaire (4-item), designed by Forrest et al (2018),^13^ assesses self-reported experiences of sleep disturbance over the past 7 days.

[Time Frame: baseline & at 2 weeks]

OM 4: Changes in time spent on different near work activities

Participants will be asked to indicate the average time in hours per day spent on each of the following activities: reading, writing, computer/PAD use, smart phone, watching TV, and playing video games.

[Time Frame: baseline & at 2 weeks]

**Interventions**

***Control group:*** *health education only (control)*

Participants will have access to online health information and stay-at-home workout videos according to government-issued recommendations. Breaks are part of the online curriculum and students will be instructed by teachers who are not aware of the study allocation to take rest and exercise breaks.

***Intervention group:*** *health education + access to the peer-to-peer live streaming application*

Participants will receive the identical health information session, online curriculum, workout videos, and breaks as indicated in the control group. Additionally, at the beginning of the study, participants will have access to a peer-to-peer live-streaming application that offers Recess and Exercise Advocacy Program (REAP). REAP is a peer-to-peer live-streaming platform that allows users to shoot short videos or photos related to their physical exercise or eye relaxation (e.g., staring out of the window) using their smartphones. The app has been optimised to be used with the iPhone or Android. When taking an online course recess, participants will have access to the REAP platform that allows them to watch stay-at-home workout videos developed by exercise physiologists. Participants will be able to create their own workout videos/photos and upload them to the live-streaming platform. When upload is completed, participants will receive instant motivational messages to increase their engagement with the program.

**Randomisation and masking**

We will conduct a cluster randomized trial, in which schools will be randomly assigned to either the intervention or control group. This study design will allow for peer-to-peer classmate support in the use of REAP, while avoiding cross arm communication and contamination. The block size is 4, and will be 1:1 assigned to the two groups. An independent statistician will perform the randomization by using an online random number generator ([http://randomization.com](http://randomization.com/)). In each school, two classes will be randomly selected from grade 7 with identical homeschool curriculum.

Participants will not be masked, due to the nature of the intervention relying on peer-to-peer support. However, the assessors will be unaware of the assigned intervention when collecting the outcome data, which will be performed by the use of electronic, self-administered questionnaires. In addition, the statistician will have no knowledge of group allocation. until completion of all analyses. To minimize the potential bias in reporting outcomes, we will inform the participating students that their responses on questionnaires are not known to their parents or teachers.

***Statistical Methods:***

## Sample size

A sample size of 12 schools for this study, a cluster randomized trial, is selected based on the following assumptions:

- The children’s anxiety score after intervention will be reduced by 2.5 points, with standard deviation of 5, and the anxiety score of the control group will not change (0 point).
- A two-sided significant level of 0.05, to achieve a power of 90%.
- The average cluster size is approximately 80 students in each school and the intra cluster correlation coefficient (ICC) within school is estimated as 0.02.^14^
- Participation rate of 90%.
- Attrition rate of 20%.

The sample size is calculated by software PASS 16.0 (NCSS, LLC, USA).

## Interim analyses and stopping rules

No interim analyses will be conducted in consideration of the low risk to the participants and the short study duration.

## Pre-specified subgroup analysis

No pre-specified subgroup analysis will be conducted.

## Statistical analysis plan

The distribution of baseline characteristics will be reported by the use of mean (standard deviation) or median (inter quartile range) for continuous variables, and frequency (percentage) for categorical variables.

The intention-to-treat analysis will be applied for both primary and secondary analyses. The unadjusted mean differences between study arms in the change after 2 weeks and 95% confidence interval (CI) for primary and secondary outcomes will be calculated using the linear regression. The adjusted intervention effect on the primary outcome and 95% CI will be estimated using linear regression adjusting for baseline measures where appropriate. The study group and all variables with p < 0.20 in the univariable regression analyses will be included in the multivariable regression analysis. A two-sided P < 0.05 will considered statistically significant. All analysis will account for the cluster effects within schools.

Histogram, Q-Q (quantile-quantile) plot will be used to check the normality assumption of t test and linear regression models. If the normal distribution is not satisfied, the normal transformation will be used.

To satisfy the intention-to-treat criteria, all missing data will be imputed. Multiple imputation will be used to impute missing data by creating 20 copies of the data, and Final results were obtained by averaging these 20 datasets using Rubin’s rules.^15^

All analysis will be performed using Stata 15.0 (Stata Corp, College Station, TX).

## Safety data analysis

Serious adverse events will not be expected given the low risk intervention.

**Results**

This study was funded by the Zhongshan Ophthalmic Center, Sun Yat-sen University, in March 2020 and received Institutional Review Board approval in March 2020. The anticipated recruitment start date for this trial is March 16 2020. The COVID-19 pandemic may cause some delay in the initiation of the trial in the recruitment sites.

References:

1. Bayham J, Fenichel EP. Impact of school closures for COVID-19 on the US health-care workforce and net mortality: a modelling study. *Lancet Public Health* 2020.Doi: 10.1016/S2468-2667(20)30082-7.

2. Mahase E. Covid-19: schools set to close across UK except for children of health and social care workers. *BMJ* 2020; **368**: m1140.

3. Wang G, Zhang Y, Zhao J, Zhang J, Jiang F. Mitigate the effects of home confinement on children during the COVID-19 outbreak. *Lancet* 2020; **395**: 945-7.

4. Yang GY, Huang LH, Schmid KL, Li CG, Chen JY, He GH, et al. Associations Between Screen Exposure in Early Life and Myopia amongst Chinese Preschoolers. *Int J Environ Res Public Health* 2020; **17**. Doi: 10.3390/ijerph17031056.

5. Lanca C, Saw SM. The association between digital screen time and myopia: A systematic review. *Ophthalmic Physiol Opt* 2020; **40**: 216-29.

6. Harrington SC, Stack J, O'Dwyer V. Risk factors associated with myopia in schoolchildren in Ireland. *Br J Ophthalmol* 2019; **103**: 1803-9.

7. Jaiswal S, Asper L, Long J, Lee A, Harrison K, Golebiowski B. Ocular and visual discomfort associated with smartphones, tablets and computers: what we do and do not know. *Clin Exp Optom* 2019; **102**: 463-77.

8. Murray R, Ramstetter C. The crucial role of recess in school. *Pediatrics* 2013; **131**: 183-8.

9. Orben A, Przybylski AK. The association between adolescent well-being and digital technology use. *Nat Hum Behav* 2019; **3**: 173-82.

10. Spence SH. A measure of anxiety symptoms among children. *Behav Res Ther* 1998; **36**: 545-66.

11. Zhao J, Xing X, Wang M. Psychometric properties of the Spence Children's Anxiety Scale (SCAS) in Mainland Chinese children and adolescents. *J Anxiety Disord* 2012; **26**: 728-36.

12. Segui MM, Cabrero-Garcia J, Crespo A, Verdu J, Ronda E. A reliable and valid questionnaire was developed to measure computer vision syndrome at the workplace. *J Clin Epidemiol* 2015; **68**: 662-73.

13. Forrest CB, Meltzer LJ, Marcus CL, de la Motte A, Kratchman A, Buysse DJ, et al. Development and validation of the PROMIS Pediatric Sleep Disturbance and Sleep-Related Impairment item banks. *Sleep* 2018; **41**. Doi: 10.1093/sleep/zsy054.

14. Negrel AD, Maul E, Pokharel GP, Zhao J, Ellwein LB. Refractive Error Study in Children: sampling and measurement methods for a multi-country survey. *Am J Ophthalmol* 2000; **129**: 421-6.
